# Health literacy and guideline-adherent lifestyle in people with chronic kidney disease: exploring factors associated with usage intention of a structured m-health program and pilot data on actual behavior change

**DOI:** 10.3389/fneph.2025.1629438

**Published:** 2025-09-03

**Authors:** Laura I. Schmidt, Mario R. Jokisch, Lea Espey, Viet Anh-Thu Hentschel, Daniela Rose, Susanne Fleig, Malte Waldeck, Jan David Best, Jürgen Wagner

**Affiliations:** ^1^ Institute of Psychology, Heidelberg University, Heidelberg, Germany; ^2^ Bavarian Center for Digital Health and Social Care, Kempten University of Applied Sciences, Kempten, Germany; ^3^ Nephrologicum Lausitz, Cottbus, Germany; ^4^ Nephrology, Endocrinology, Diabetology and Dialysis (NEDD*Grünstadt), Grünstadt, Germany; ^5^ Department of Nephrology and Clinical Immunology, Medical Faculty, RWTH Aachen, Aachen, Germany; ^6^ Rheinisch-Westfälische Technische Hochschule (RWTH) Aachen University, Aachen, Germany; ^7^ Center for General Medicine and Geriatrics, University of Mainz, Mainz, Germany; ^8^ MediClin Staufenburgklinik, Durbach, Germany

**Keywords:** chronic kidney disease, technology acceptance, health literacy, patient activation, behavior change, m-health, e-health, digital intervention

## Abstract

**Background:**

Although medical guidelines for chronic kidney disease (CKD) clearly recommend measures such as blood pressure control, dietary changes, regular physical activity, and consistent medication adherence, individuals frequently encounter challenges in implementing these behavioral modifications. In medical practices, there is a lack of time and resources to comprehensively support CKD patients and low-threshold (digital) interventions aimed at enhancing patient activation are needed. This paper analyzes the acceptance and usage intention (Study 1) and the contribution to health literacy and behavioral change (Study 2) of a m-health program for CKD (“Oska”). The Oska program combines personal counseling via video calls with app-based support and is theoretically grounded in the Health Action Process Approach (HAPA), with a strong emphasis on fostering self-efficacy and promoting implementation in daily routines.

**Method:**

Study 1: An online survey was conducted with *N* = 401 individuals with CKD and/or hypertension, obesity, type 2 diabetes, or coronary heart disease (age: 50–89 years, *M* = 64.1, 49% female). Participants were recruited via the provider Appinio and presented with a vignette illustrating the Oska program and answered questionnaires on usage intention, desired support, compatible health benefits, health literacy, and perceived usefulness. Study 2: *N* = 109 participants with CKD, who already took part in the Oska program for an average of 4.7 months (age: 29–84 years, *M* = 62.3, 64% female, BMI: *M* = 29.6), completed established questionnaires on working alliance, kidney-specific health literacy, and behavior change. The analysis was conducted using structural equation models and linear regression analyses.

**Results:**

Acceptance and usage intention in study 1 were high and predominantly explained by compatible health benefits, health literacy, and perceived usefulness, but largely independent of sociodemographic factors and health-related variables. In study 2, higher health literacy was primarily fostered by longer program participation and, most notably, by a positive trust relationship (working alliance) (*R²_adj_* = .48) Successful behavior change (across all guideline areas) was primarily attributed to a positively evaluated working alliance and Oska’s contribution to health literacy, rather than sociodemographic factors or the number and type of diagnoses (*R²_adj_* = .14).

**Discussion:**

Digitally delivered coaching combined with app-based support is not only acceptable but may be particularly effective for CKD patients with low health literacy and multiple comorbidities. Relevant determinants include a trusting coaching relationship and a focus on health literacy as well as self-efficacy in implementing measures in everyday life.

## 1 Introduction

Caring for individuals with chronic diseases represents a significant challenge for the entire healthcare systems worldwide and also accounts for approximately 70% of healthcare expenditures in Germany. Around 53% of the German population aged 40 years and older lives with two or more chronic conditions (1). With each additional chronic disease, healthcare costs increase exponentially.

Particular attention is needed for individuals with chronic kidney disease (CKD), who now make up 10% of the German population. Statistically, they exhibit the highest number of comorbidities, with more than 80% to even 98% having a secondary condition (2, 3), especially diabetes, hypertension, and heart failure. CKD is an independent cardiovascular risk factor, and cardiovascular risk increases linearly with CKD stage (4). In comparison, for diabetes, depression, or cancer, individuals are more likely to only have the primary condition, which emphasizes the importance and complexity of proactive disease management (2). On average, annual healthcare costs per person with CKD amount to around €9,300—largely driven by preventable hospitalizations and cardiovascular complications. This pattern is consistent worldwide, with projections indicating a substantial increase in the coming years (5). These trends, accompanied with robust findings that declining kidney function is associated with deteriorating quality of life in people with CKD (6) underscore the urgent need for earlier diagnosis and proactive disease management to mitigate disease progression.

### 1.1 Low awareness and low adherence to clinical guidelines and recommendations

Although clinical guidelines [i.e., KDIGO; (7)] clearly recommend early secondary prevention measures such as blood pressure control, dietary changes, regular physical activity, and consistent medication adherence, many individuals with CKD struggle to implement these behavior changes in the long term: In large German cohort studies, 50% do not take their medication regularly, and 80% fail to maintain necessary lifestyle changes such as reducing salt intake or increasing physical activity (8–10). In medical practices, there is often a lack of time and resources to provide CKD patients with comprehensive support. The average consultation length in general practices is less than 10 min in Germany (*M* = 7.6 min, *SD* = 4.3), although, according to various sources, people with multiple chronic conditions often spend 10 to 15 minutes in consultations (11–13). International data are even showing shorter time frames, with 50% of the world population having only 5 minutes or even shorter consultations with their physician (12). Accordingly, it becomes evident that the available consultation time is limited to addressing immediate medical necessities, leaving insufficient capacity for comprehensive lifestyle counseling. Moreover, people with CKD pose a particular challenge: even when diagnosed, individuals are often unaware of their kidney condition, which may be due to documentation issues in general practices, asymptomatic early stages, or communication gaps because of perceived low clinical urgency or simply omission during time-pressured doctor visits (14, 15). This is associated with limited disease-specific knowledge or understanding of effective self-management strategies. But also, when awareness and basic health literacy are present, lifestyle changes recommended by guidelines are rarely implemented—despite first evidence that structured behavior change interventions can significantly improve relevant health outcomes (16–18).

This raises the question of whether low-threshold (digital) interventions for individuals with CKD—ideally accessible with basic technological skills and available free of charge—can effectively support patient activation and promote lifestyle modifications. Over the past decade, significant advancements in mobile technology have created new opportunities for the digital delivery of healthcare services. The development and implementation of digital health interventions aimed at enhancing self-management and promoting health-related behaviors have been notably accelerated by the COVID-19 pandemic, which highlighted the need for scalable, remote care solutions. However, in the context of early, pre-dialysis kidney disease, theory-driven approaches based on models from health psychology and behavior science are very scarce. To the best of our knowledge, there is only one other systematically developed theory- and evidence-based digital self-management program (“My Kidneys & Me”) for people with non-dialysis CKD yet (19, 20). Partially positive outcomes have been reported regarding increased patient activation (measured with the PAM-13), particularly among participants with initially low activation levels. However, data on program acceptance has not been reported. The My Kidneys & Me intervention targeted a relatively homogeneous group (CKD stages 3 and 4) and was conducted in secondary care. This may partly limit generalizability, especially considering that the majority of individuals with CKD are managed in primary care settings (e.g., GPs), where awareness of the disease and its implications is often even lower.

### 1.2 Potential of digital interventions for CKD and related diseases, but questionable acceptance?

From a public health perspective, e-health and m-health have gained interest, with the central role of health insurance providers in improving care for individuals with chronic illnesses increasing in importance. Rather than acting solely as cost reimbursers, they are now, in most countries, legally empowered to actively participate in care management by offering continuous support to policyholders, enhancing health literacy, and helping to prevent complications through behavior change interventions. In Germany, the legal framework for such involvement is provided by the German Social Code Book V (Sozialgesetzbuch Fünftes Buch – SGB V, 2025) (21), which governs statutory health insurance and defines the benefits and organizational structure of the healthcare system. Under § 140a SGB V, health insurers are authorized to enter into selective contracts with providers of innovative health services, allowing them to expand their range of services beyond the standard statutory care (“Regelversorgung”). In addition, §§ 68b and 25 SGB V permit health insurers to analyze insured persons’ health data to identify individuals who might benefit from targeted interventions. This enables insurers to proactively offer personalized, data-driven care programs tailored to the specific health needs of their members. This could enable a transition from passive payer to active health manager and could contribute to addressing existing gaps in care, but only, if people with chronic diseases, typically in a higher age range, are willing to adopt the digital offerings. To date, the opportunity to develop digital interventions and systematically integrate them into existing medical care (i.e. primary care) pathways remains largely underutilized (22). Moreover, as especially individuals with multiple chronic conditions often report low perceptions of control over the course of their diseases and low self-efficacy to initiate respective actions (23), it is questionable, how they would evaluate, accept, and intend to use digital coaching programs designed to assist with condition-specific health literacy and guideline-adherent behavior change. Moreover, although Germany has established nationally standardized Disease Management Programs (DMPs) for chronic conditions such as type 2 diabetes, coronary heart disease, and COPD, no such program currently exists for chronic kidney disease (CKD), either in analog or digital form. To explore acceptance, we draw on extensions of the Technology Acceptance Model [TAM; (24)] and the Unified Theory of Acceptance and Use of Technology [UTAUT; (25)]. While digital health applications hold significant potential for both individual and public health, acceptance among older adults remains a critical barrier. TAM and UTAUT posit that perceived usefulness and ease of use predict usage intention, yet empirical studies applying both in older populations—particularly those over 75—are limited. Scarce evidence suggests that in the ‘young-old’ (60–75 years), perceived usefulness is key, whereas in older age, self-efficacy and technology-specific factors such as compatibility and trust become more important (26–29) with ease of use declining in relevance (30). We therefore aimed to address both perceived usefulness and compatible health benefits in order to predict the intention to use a digital coaching program. Compatible health benefits capture how well participants’ values and needs corresponded to the respective (digital) service or program and serve as an indicator of compatibility, which is outlined in the Diffusion of Innovations Theory [DOI; (31)]. More closely, compatibility is defined by the degree to which an innovation (in our case: an m-health program) is perceived as being consistent with an individual’s existing values, past experiences, and needs (21). If the m-health program aligns well with these factors, it is considered highly compatible and more likely to be adopted. We further aimed to explore the role of health-related factors such as the degree of desired support in disease-related areas and participants’ health literacy. Health literacy is defined as the ability to access, understand, appraise, and apply health-related information, enabling informed decisions and actions in everyday health contexts (32) and has been shown to affect the acceptance of digital health technologies (33, 34).

### 1.3 Digital intervention in the case of chronic kidney disease: the Oska program

**Figure 1:**
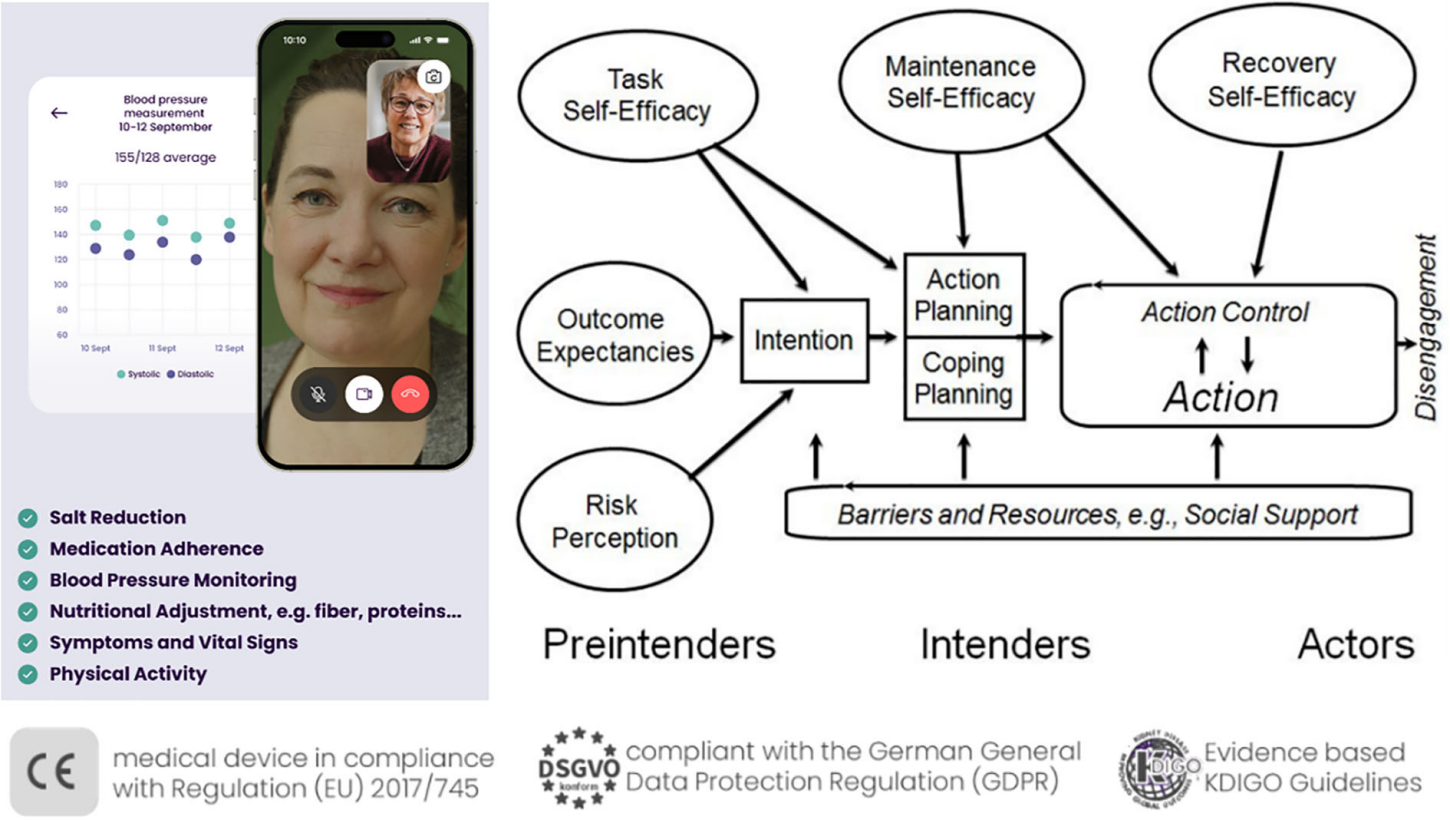
The Oska program, personal, app-supported coaching on the basis of the Health Action Process Approach. Right part of the figure, Health Action Process Approach [HAPA, adapted from Schwarzer (35)].

In health promotion efforts targeting populations with limited awareness of their medical condition such as (early-stage) CKD, stage-based behavioral models present a promising framework by facilitating the design of interventions that are appropriately tailored to individuals’ respective stages of readiness for change. Thereby, also participants without awareness, motivation, or health-related intentions, which might be driven by asymptomatic early stages or communication gaps in healthcare, could be engaged at their current stage or level of understanding. The present m-health program, the *Oska program* (https://www.oska-health.com/), therefore draws on the Health Action Process Approach [HAPA; (35, 36)], which is a widely recognized and empirically supported stage model that has successfully been applied in lifestyle interventions among people with chronic diseases [e.g., (36)]. However, with focus on chronic kidney disease, there is only the approach of the program My Kidneys & Me among patients from secondary care hospital sites across England that includes elements of the HAPA (i.e., risk awareness, outcome expectancies). The HAPA model differentiates between two key phases of health behavior change: the *motivational phase*, in which individuals have not yet formed a specific intention to act (*Preintenders*, see **Figure 1**), and the *volitional phase*, during which existing intentions are translated into concrete behavior through planning strategies, as well as the identification of barriers and personal resources (*Intenders*, **Figure 1**). A key reason for selecting the Health Action Process Approach (HAPA) over other prominent models of health behavior change – such as the Theory of Planned Behavior [TPB; (37)] or the COM-B model (38) – was its strong emphasis on the implementation of behavior change in daily routines, particularly highlighted in its volitional phase. Additionally, the HAPA offers a more parsimonious framework by distinguishing only between preintenders and intenders, allowing for more targeted intervention strategies. In contrast, other stage models, such as the Transtheoretical Model [TTM, with five stages; (39)] and the Precaution Adoption Process Model [PAPM, with seven stages; (40)], have been criticized for relying on arbitrary staging algorithms and for lacking consistent empirical evidence supporting the effectiveness of stage-matched interventions across their numerous stages (41).

In the present work, following successful patient enrolment, the Oska program initiates a structured, evidence-based intervention designed to promote sustainable lifestyle modification. The program is delivered via video calls with personal certified *health coaches* (i.e., registered nurses with specialized training in nephrology and university-trained dietitians), and the Oska app supports with informational chapters and tracking tools for the parameters of interest (e.g., blood pressure, physical activity, and nutrition diaries). Moreover, live webinars are offered every two or three months including topics such as sodium or protein intake, medications, physical activity advice and sessions for different fitness levels (42), or principles to facilitate habit formation (43).

At the outset, the health coaches target motivational determinants by supporting participants in developing a better understanding of their chronic kidney condition and its interaction with common comorbidities such as hypertension, diabetes mellitus, and cardiovascular disease. Beyond the mere transmission of knowledge, the intervention emphasizes the enhancement of self-efficacy—that is, individuals’ confidence in their ability to positively influence health outcomes through their own behavior. A key objective at this stage is to increase participants’ outcome expectancies, helping them recognize the effectiveness of their actions in managing disease progression.

The Oska model incorporates the concept of patient activation, which has been shown to significantly improve adherence and long-term behavioral change (16, 19). Psychological barriers, including maladaptive beliefs (e.g., “There’s nothing I can do about my kidney disease”, “At my age, exercise—particularly strength training—is no longer beneficial”), are addressed using evidence-based motivational interviewing techniques (44). For example, maladaptive beliefs are targeted by respective information via the app chapters and webinars, but also via resource communication by the health coaches in the video calls that focus on simple measures in order to heighten outcome expectancies. For example, the approach of the ‘Lifestyle-integrated Functional Exercise’ (LiFE) program (45) with easy and adaptable exercises in the homes is introduced by the coaches for physically inactive older individuals or those with higher risk of falling. Moreover, techniques of Motivational Interviewing such as eliciting change talk (i.e., “What would be some of the benefits if your belief turned out to be incorrect?”) are used, alongside reflective listening, double sided reflection, affirmation and carefully developing discrepancy (between beliefs and goals/motives).

All health coaches have completed a certified advanced training in Motivational Interviewing that lasted a total of 9 full days, or have completed full training as systemic coaches, which includes these elements. In addition, there is a daily opportunity each morning in an internal online meeting of the health coaches and a psychologist to anonymously discuss individual cases within the team, and once a week a training session takes place featuring a concrete case example related to topics from Motivational Interviewing. Throughout the process, participants receive continuous, individualized guidance to progressively build their readiness for change. Upon achieving sufficient motivational readiness, a concrete health behavior goal is collaboratively defined during the next one-on-one video consultation. This goal-setting and planning phase follows the established 5A framework [Assess, Advise, Agree on goals, Assist, Arrange follow-up; (46)].

A central pillar of the program is the focus on a strong trust-based relationship between coach and participant. The so-called working alliance has been widely recognized as a key concept in psychotherapy and coaching processes. However, to the best of our knowledge, it has not yet been an explicit focus in m-health programs aimed at patient activation and health behavior change. In the theoretical model and in respective questionnaires, a strong working alliance is built on three factors, namely a strong bond, agreement on goals, and agreement on tasks and processes in the coaching process (47). A positive and strong working alliance has been shown to robustly predict subsequent treatment outcomes (48, 49).

More detailed (medical and behavioral) outcomes are currently investigated in a larger longitudinal study over the course of six to nine month of participation in an independent sample of participants that did not take part in one of the two studies presented here.

### 1.4 Research questions: usage intention and first behavioral outcomes of an m-health program for CKD

In the two present studies, our first aim was to explore factors for acceptance and usage intention of an m-health intervention program among the target group of people aged 50+ with either a CKD diagnosis or a “probable” CKD, namely with (combined) diagnoses of hypertension, cardiovascular disease, diabetes, and obesity. To date, there is insufficient evidence as to whether a free health insurance–funded program offer can effectively motivate older individuals with (presumed) chronic kidney disease to engage in such m-health interventions. Our second aim was to present first pilot data of an independent sample of program participants in a real-world setting, namely non-dialysis CKD patients that were enrolled in the Oska program after receiving the offer from their health insurance provider.

As literature on acceptance of m-health programs for chronic diseases is scarce, more insights are needed of how best to reach these individuals and motivate them to participate in relevant programs and health insurance offerings. Moreover, actual data on behavior change and the relationship with kidney specific health literacy among people with CKD are largely missing (19).

More specifically, in Study 1, we aimed to evaluate using structural equation modeling whether intention to use the Oska m-health program, offered in a realistic, insurance-based scenario, can be predicted by core acceptance-related constructs: perceived usefulness, compatible health benefits, and health literacy. Based on TAM, UTAUT, and DOI, we assumed that usefulness and compatibility would be central drivers of intention, while health literacy may act either as a facilitator or, in line with recent findings, as a compensatory factor particularly relevant among individuals with lower prior knowledge. The role of desired support in disease-related domains was examined as an additional motivational factor. Sociodemographic and health-related variables (age, gender, number of diseases, and subjective health status) were included to assess their potential explanatory value relative to these psychological predictors.

Our research questions in Study 1 were: To what extent do perceived usefulness, compatibility, and health literacy predict intention to use an m-health program for CKD in a real-world insurance-based setting? What is the role of desired support in disease-related domains in shaping motivation to adopt m-health interventions? How do sociodemographic and health-related variables compare to psychological predictors in explaining intention to use the program? Study 2 focused on participants’ experiences with the Oska program in a real-world setting after at least four weeks of participation. We explored their perceptions of the program’s usability, the coach–patient relationship, and the extent of gains in kidney-specific health literacy and self-reported behavior changes in line with KDIGO guidelines. Particular attention was given to the perceived quality of the working alliance with their health coach, which—based on the Health Action Process Approach [HAPA; (36)], was considered a central mechanism for fostering motivation and successful implementation. We also examined how individual characteristics and the duration of program participation were associated with the reported outcomes. With this exploratory study, we aimed to contribute to the scarce literature on theory-based m-health interventions for individuals with chronic kidney disease.

Our research questions in Study 2 were: How do participants perceive the usability of the Oska m-health program and the quality of the coach–patient relationship after at least four weeks of use? Does participation in the program lead to improvements in kidney-specific health literacy and behavior changes consistent with KDIGO guidelines? How does the perceived quality of the working alliance with the health coach relate to health literacy, motivation, and implementation of health behaviors?

Both studies were part of a larger project at Heidelberg University called “Self-Management and Behavior Change in Chronic Kidney Disease (CKD): A Complement to Medical Care” (Registration Open Science Framework: https://osf.io/7dup9). The studies followed APA ethical standards as well as the 1964 Helsinki declaration and its later amendments. The ethics commission of the Faculty of Behavioral and Cultural Studies at Heidelberg University, Germany, obtained ethical approval (protocol number: AZ Schm 2024 1/1).

## 2 Study 1: factors explaining the intention to use an m-health program (“Oska”) among individuals with (probable) chronic kidney disease

### 2.1 Method study 1

#### 2.1.1 Procedure and sample description study 1

A total sample of 401 adults was recruited via Appinio, a German market research institute in September 2024. To be included, participants had to be at least 50 years of age and had to have a CKD diagnosis or at least one chronic condition associated with an increased risk of developing or already having kidney disease (i.e., hypertension, obesity, type 2 diabetes, and/or heart disease). These criteria were applied to align the study sample as close as possible to the main target audience of the Oska program (CKD or highly probable diagnosis, even in the absence of awareness). Alongside CKD and the four inclusion diagnoses, participants were able to select up to 12 diagnostic categories. Outlier analyses were performed for all study variables. One participant was labeled as an outlier as he selected all 12 possible diagnoses and was excluded. One other participant was identified due to a longer completion time but as the results did not change with this person, we decided not to exclude this person.

The final sample of *N* = 400 participants had a mean age of 64.11 years (*SD* = 8.18, range = 50–89), 49% were women and 51% were men. All participants resided in Germany, most of them (67%) living in rural areas. On average, participants reported having 1.89 chronic diseases (*SD* = 1.05, range = 1–6). The self-reported disease with the highest prevalence was hypertension (72%), followed by obesity (38%), type 2 diabetes (29%), and heart disease (18%). Less than 10% of the sample stated having other cardiovascular or renal diseases. Participants first read a short vignette on the Oska program and its benefits (e.g., regular video calls and chats with healthcare professionals, app features for documenting health records, informational chapters on CKD and related chronic diseases, counseling on health promoting activities) and then completed an online questionnaire with the following scales.

#### 2.1.2 Measures study 1

*Intention* to use the Oska program was measured with two items (“How likely are you to try the Oska program if it were provided free of charge by your health insurance provider?” and “The Oska program is of personal interest to me”), based on Davis et al. (24). They were answered on a Likert scale ranging from 1 (“very unlikely/strongly disagree”) to 6 (“very likely/strongly agree”).

For *desired support*, participants were asked whether or not they wished for more support in five disease-related areas (e.g., “how my medication affects my health”, “how nutrition affects my health”). The sum score was interpreted as the degree of desired support.

To assess *compatible health benefits* [self-developed, adapted from (31)], participants were given a list with six health-related aspects, all implemented by the Oska program (e.g., “access to reliable health information, such as evidence-based content on kidney health”, “receiving feasible health-related tips, such as customized advice, recipes, suggestions concerning physical activity). They were instructed to rate how important these aspects were to them on a Likert scale from 1 (“not important at all”) to 6 (“very important”).

*Health literacy* (adapted from 32) was assessed with seven items (e.g., “I feel that I am well informed about my state of health”, “I don’t know how my medication works (–)”), using a Likert scale from 1 (“strongly disagree”) to 6 (“strongly agree”). We opted for a general measure (not CKD specific), as not all participants were diagnosed with CKD in Study 1.

The measurement of *perceived usefulness* was adapted based on Davis et al. (24). Participants were given a list with eleven features of the Oska program (e.g., “personal assistance provided by health counselors”, “documenting health records”) and indicated for each item whether or not they saw it as useful for themselves. The sum score was interpreted as the degree of perceived usefulness.

Participants’ *subjective health status* was assessed with one item (“How would you rate your current health?”), scored on a Likert scale from 1 (“I am very ill”) to 10 (“I am absolutely healthy”). Almost all scales yielded good to excellent internal consistency (intention: Spearman-Brown coefficient = .93, compatible health benefits: α = .90, health literacy: α = .84, perceived usefulness: α = .91). Desired support demonstrated moderate internal consistency (α = .68). For perceived usefulness and desired support, Cronbach’s alpha was calculated based on tetrachoric correlations due to the scales’ dichotomous response type (50).

#### 2.1.3 Statistical analyses study 1

Structural equation modeling was conducted using the lavaan package in Rstudio (Version 2023.06.1 + 524). Intention to use the Oska program served as the dependent variable and was entered as a latent factor with two items. Desired support, compatible health benefits, health literacy, and perceived usefulness were included as latent predictor variables measured by five, six, seven, and eleven items, respectively. Age, gender, subjective health status, and the number of chronic diseases were entered as predictor variables to adjust for their effects. They were included as manifest variables indicated by one item each. A CFI score ≥ 0.95 and a RMSEA score ≤ 0.06 were interpreted as indicators of a good model fit (51). The analysis was performed using the weighted least squares mean and variance adjusted (WLSMV) estimator since perceived usefulness and desired support were indicated by categorical items (52).

### 2.2 Results study 1

The overall intention to use the Oska program was high (*M* = 4.24, *SD* = 1.54, possible scores from 1 = very unlikely/strongly disagree to 6 = very likely/strongly agree). Descriptive statistics and correlations for all study variables are shown in **Table 1**. Additionally, a t-test for independent samples revealed that participants’ intention to use the Oska program did not significantly differ between those residing in rural and those residing in urban areas (*t*(280) = 1.48, *p* = .139).

**Table 1:** Descriptive statistics and correlations for study 1.

Variable	*M*	*SD*	1	2	3	4	5	6	7	8	9
1. Age	64.11	8.18	—	.10*	.08	.06	-.14**	-.04	.00	.25***	-.06
2. Gender (female %)^a^	49.25	—		—	.03	.11*	.01	.06	-.03	-.01	-.07
3. Number of diseases	1.89	1.05			—	-.22***	.08	.22***	.12*	-.16**	.21***
4. Subjective health^b^	5.8	2.1				—	-.01	-.04	.03	.14**	-.03
5. Intention^c^	4.24	1.54					—	.50***	.67***	-.49***	.53***
6. Desired support^d^	2.02	1.45						—	.46***	-.41***	.52***
7. Compatible health benefits^c^	4.73	0.9							—	-.39***	.45***
8. Health literacy^c^	3.64	1.08								—	-.27***
9. Perceived usefulness^e^	4.2	3.22									—

*N* = 400.

^a^Men = 0, women = 1. ^b^1 = “I am very ill” to 10 = “I am absolutely healthy”. ^c^1 to 6, higher scores indicate stronger agreement. ^d^0 to 5, higher scores indicate stronger agreement. ^e^0 to 11, higher scores indicate stronger agreement.

*p <.05, **p <.01, ***p <.001.

#### 2.2.1 Structural equation model explaining the intention to use the Oska program

**Figure 2:**
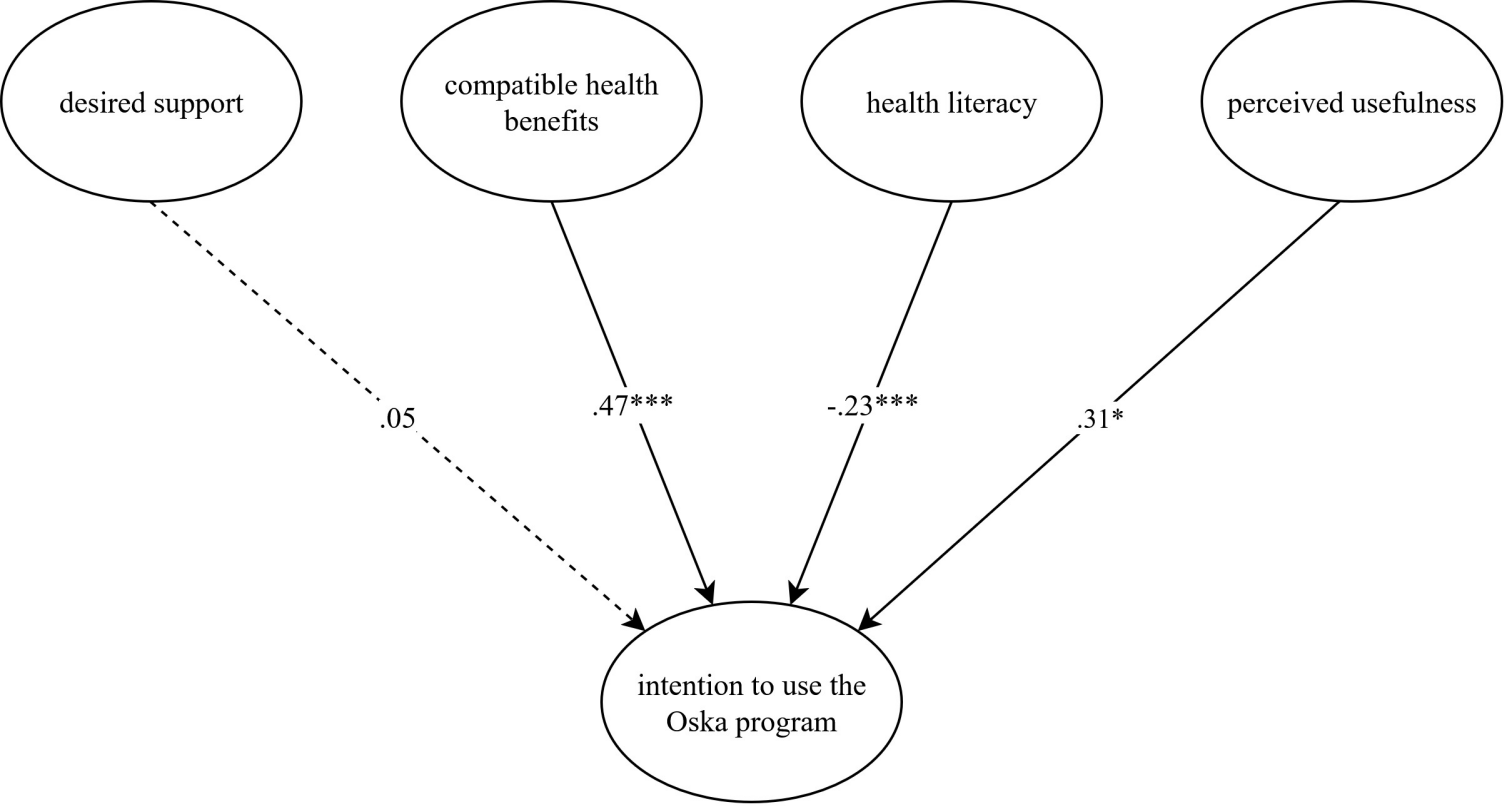
Latent structural equation model explaining the intention to use the Oska program. *N* = 400. The model is presented with standardized path coefficients. Compatible health benefits capture how well participants’ values and needs corresponded to the respective (digital) service or program and serve as an indicator of compatibility, which is outlined in the Diffusion of Innovations Theory [DOI; Rogers (31)], perceived usefulness and usage intention are core concepts of the Technology Acceptance Model [TAM; Davis et al. (24)] and the Unified Theory of Acceptance and Use of Technology [UTAUT; Venkatesh et al. (25)]. Health literacy and desired support were added to analyze their additional value in our study. Age, gender, subjective health status, and number of chronic diseases served as control variables and were entered with direct paths to intention. Although included in the analysis, they were omitted from the figure to improve legibility. This figure was created with the online software draw.io (Version 27.0.2). *p < .05, ***p < .001.

An initial analysis of the measurement model yielded satisfactory fit indices (CFI = .991, RMSEA = .047). In a second step, we evaluated the final latent structural equation model. Results are presented in **Table 2**. The model demonstrated a good fit (CFI = .990, RMSEA = .044). Desired support, compatible health benefits, health literacy, perceived usefulness, and the control variables accounted for 74.3% of the variance in the intention to use the Oska program. The model’s standardized path coefficients are displayed in **Figure 2**.

**Table 2:** Results of the latent structural equation model explaining the intention to use the Oska program.

Path	*b*	β	*SE*	*p*
Intention^a^ ← Desired support^b^	0.116	.051	.432	.789
Intention ← Compatible health benefits^a^	0.655	.467	.079	<.001
Intention ← Health literacy^a^	-0.307	-.233	.089	.001
Intention ← Perceived usefulness^c^	0.400	.305	.170	.019
Intention ← Age	-0.007	-.060	.004	.096
Intention ← Gender^d^	0.057	.062	.054	.293
Intention ← Number of diseases	-0.086	-.099	.037	.020
Intention ← Subjective health status^e^	0.004	.010	.016	.781

*N* = 400.

^a^1 to 6, higher scores indicate stronger agreement. ^b^0 to 5, higher scores indicate stronger agreement. ^c^0 to 11, higher scores indicate stronger agreement. ^d^Men = 0, women = 1. ^e^1 = “I am very ill” to 10 = “I am absolutely healthy”.

In line with our assumptions, compatible health benefits (β = .467, *p* = <.001), health literacy (β = −.233, *p* = .001), and perceived usefulness (β = .305, *p* = .019) were significantly associated with intention. Contrary to our expectation, the relationship between health literacy and intention was negative. In other words, participants who reported a lower degree of health literacy stated a higher level of intention to use the Oska program. Other than expected, there was no significant association between the degree of desired support and intention (β = .051, *p* = .789). The sociodemographic and health-related variables did not significantly predict intention, except for the number of chronic diseases which exerted a small negative effect (β = −.099, *p* = .020). However, the effect was not reflected in the correlation of the variables. We therefore suspect that this might be an incidental finding and refrain from an interpretation.

A *post-hoc* analysis was conducted to further explore the non-significant association between the degree of desired support and intention to use the program. Taking into account the strong correlation between desired support and perceived usefulness (*r* = .52), the model was performed without including perceived usefulness as a predictor. The revised model revealed a significant relationship between the degree of desired support and intention (β = .322, *p* = .007), suggesting that desired support and perceived usefulness may share a substantial proportion of the explained variance in intention.

### 2.3 Discussion study 1

Participants generally reported a strong usage intention, confirming the high relevance of the Oska program to individuals with either diagnosed CDK or a probable CKD due to frequent comorbid diagnoses. Structural equation modeling revealed that perceived usefulness significantly predicted participants’ intention to use the program. This finding is in line with TAM (24) and UTAUT (25) and adds to previous studies suggesting that perceived usefulness might be a relevant factor in explaining older adults’ intention to utilize healthcare technologies (27, 28). Moreover, our data indicated that perceived compatible health benefits were a major predictor of participants’ usage intention, which is consistent with DOI (31) and findings reported by Harris and Rogers (27). We observed a negative association between participants’ health literacy and their intention to use the Oska program. This suggests that the program, as described in the vignette, was particularly appealing to individuals who face challenges in accessing and understanding health information. Similarly, Lightfoot et al. (20) reported that their digital health intervention for individuals with non-dialysis chronic kidney disease (CKD stages 3 & 4) was most effective among those with low levels of patient activation. In this context, it is encouraging that also participants with lower health literacy expressed high (or even a higher) intention to use the Oska program, as this population may especially benefit from a structured e-health intervention. Furthermore, our analysis indicated that usage intention was largely unaffected by participants’ age, gender, or subjective health status. However, since we did not include measures of education or income, we recommend that future studies incorporate these variables to gain a more comprehensive understanding.

All hypotheses were evaluated in a large sample of individuals aged 50+ that are highly prone to CKD or already have a CKD diagnosis. Hence, participants were part of the audience intended by the Oska program, which is an important strength of the present study. Our sample for Study 1 was very similar in age to the intended target group of the Oska program, since CKD is more prominent in older age groups. Ecological validity was further ensured by a realistic study procedure with participants reading a short vignette on the Oska program, just as they would when actually being informed about the Oska program by their health insurance provider.

However, several limitations must be acknowledged. First, we did not capture actual usage behavior and relied on the usage intention instead, which is a good but not a perfect indicator of subsequent technology use (53). Second, due to the cross-sectional nature of the data, we were unable to establish causal relationships between the predictor variables and usage intention. A longitudinal design with a waitlist control group is being implemented in a larger ongoing study within the same project. Nevertheless, future research should aim to link acceptance data, as in Study 1, with actual usage and behavioral data, as in Study 2, using a longitudinal design involving the same group of individuals. This approach would enable comparisons between later users and non-users and allow for more robust causal inferences. Third, recruitment via the online panel provider Appinio may have introduced a selection bias, as participation required a basic level of digital literacy. It is therefore likely that the sample consisted of individuals with greater familiarity and confidence in using digital technologies than typically found in the broader target population. Fourth, we relied on self-reported diagnoses and, due to the low awareness of CKD, do not know how many individuals actually have a confirmed CKD diagnosis. However, as we did not limit our study sample to individuals with confirmed CKD but included those with important comorbidities as well (i.e., hypertension, obesity, type 2 diabetes, and/or heart disease), we believe that we included a population that fits the target sample of the Oska program.

## 3 Study 2: pilot data on working alliance, health literacy, and actual behavior change among participants of the Oska M-Health-Program for chronic kidney disease

### 3.1 Method study 2

#### 3.1.1 Procedure and sample description study 2

*N* = 109 participants of the Oska program (age range: 29–84 years, *M* = 62.3, *SD* = 9.0, 64% female, BMI: 29.6, SD = 6.7) who had been enrolled in the program for at least one month (average duration: 4.7 months), completed questionnaires in the winter of 2024/2025. All participants had at least completed two video calls via the Oska app with their health coach, namely one longer onboarding call and at least one follow up call. Moreover, all participants had access to the tracking tools of the app for weight and blood pressure, as well as general chapters on kidney health plus individually assigned chapters on their respective comorbidities. 83% were publicly insured, and 17% had private health insurance. 10% of the originally contacted participants (*N* = 12 out of 121) did not respond to the study invitation. There were no differences in sociodemographic variables and comorbidities between those who participated and those who did not respond to the study invitation (p <.05). However, due to the small group size of the non-participants, these comparisons are slightly underpowered. Participants had on average 2.1 chronic conditions (*SD* = 1.3) with a range from one (= only CKD) to six. All participants provided informed consent. In addition to CKD, the most common comorbidities among participants were hypertension and diabetes. Inclusion criteria (also applicable to the Oska program, i.e., regardless of study participation) were: Age > 18 years and having a diagnosis of CKD, as documented by the health insurance provider. Exclusion criteria (also applicable to the Oska program) were: Requirement for dialysis, cognitive impairments (e.g., dementia) that significantly limit the ability to engage in conversations or comprehend program and app content, insufficient proficiency in the German language. The data were primarily analyzed using correlation analyses, group-comparisons, and hierarchical linear regression models. All relevant assumptions with respect to regression analyses were met (linear relationship, multivariate normality, no multicollinearity or auto-correlation, homoscedasticity). Power analyses with G*Power (54) demonstrated that the sample size of *N* = 109 was large enough to detect medium-sized effects in linear multiple regressions (fixed model, sensitivity analysis, R² deviation from zero: 2 tested predictors for the dependent variable health literacy, required effect size: f² = .12; and 4 predictors for the dependent variable behavior change, required effect size: f² = .15).

#### 3.1.2 Measures study 2

The so-called *working alliance*—defined as the bond between participants and their Oska health coach, the collaborative approach, and agreement on goals was assessed with three items (Cronbach’s α = .73) based on the Working Alliance Inventory (47, 49). Example items are: “I feel that the health coach will help me achieve the changes I desire” or “The contact with the health coach is pleasant and based on trust” on a scale from 1 (strongly disagree) to 5 (strongly agree).

*Health literacy* (with kidney specific focus) was assessed with four items (Cronbach’s α = .79) derived and adapted from the PAM-13 (55). E.g., “by participating in the Oska program, it becomes clearer what I can do myself to support my kidney health” or “the Oska program makes it easier for me to assess which everyday habits (e.g., drinking and eating habits, physical activity, etc.) are related to kidney health” on a scale from 1 (strongly disagree) to 5 (strongly agree).

Self-reported *behavior changes* in areas relevant for CKD were assessed with seven single items each regarding medication adherence, blood pressure monitoring, diet change, salt reduction, weight reduction, physical activity, stress reduction/reduction of uncertainty in the context of CKD. For each item, e.g., “The Oska program makes it easier for me to stick to my medication schedule.” Or “Since participating in the Oska program, my salt intake has significantly decreased.” Participants had the option to answer either 1 = “not relevant for me”, 2 = “yes, I would like to address that specifically now” (= Intender according to HAPA) or 3 = “yes, I already achieved noticeable positive changes with the Oska program”.

*App usability* was measured with a single item from the Telehealth Satisfaction Questionnaire for wearable technology TSQ-WT (56), namely “The Oska app is easy to use” with scores from 1 (strongly disagree) to 5 (strongly agree).

*The likelihood of recommendation* and customer loyalty was assessed via the Net Promoter Score (NPS), a market research metric, that asked about the likelihood of recommendation on a scale of 0 to 10 and is calculated by subtracting the percentage of detractors (scores 0-6) from the percentage of promoters (scores 9-10). Passives (scores 7-8) are not included in the calculation (57). Alongside this calculation that is mainly used in market research, we apply this scale as a continuous measure from 0 = extremely unlikely to 10 = extremely likely.

### 3.2 Results study 2

Correlations of the main study variables are depicted in **Table 3**.

**Table 3:** Descriptive statistics and correlations for study 2.

Variable	*M*	*SD*	1	2	3	4	5	6	7	8	9
1. Age	62.27	9.04	—	-.19	-.09	-.10	-.15	-.22^*^	-.18	.13	.16
2. Gender (female %)^a^	64	—		—	-.03	-.05	.36^**^	.25^*^	.20^+^	-.10	.04
3. Body Mass Index (BMI)	29.60	6.71			—	.12	.05	.02	.04	.33^**^	.05
4. Program Duration (month)	4.68	2.81				—	.00	.16^+^	.26^**^	.23^*^	.10
5. Usability^b^	4.49	0.68					—	.28**	43^***^	.00	.31^***^
6. Working Alliance^b^	4.48	0.50						—	.68^***^	.30^**^	.49^***^
7. Health Literacy (CKD specific)^b^	4.33	0.51							—	.32^***^	.59^***^
8. Behavior Change (Sum)^c^	14.77	3.23								—	.39^***^
9. Program recommendation^d^	9.36	0.83									—

*N* = 109.

^a^Men = 0, women = 1. ^b^1 = “strongly disagree” to 5 = “strongly agree”. ^c^1 to 6, higher scores indicate stronger agreement. ^c^Sum of seven items, each item is scored on a scale from 0 = “not relevant for me”, 1 = “yes, I would like to address that specifically now”, and 3 = “yes, I already achieved noticeable positive changes with the Oska program”. ^d^0 = “extremely unlikely” to 10 = “extremely likely”.

^+^p <.10, ^*^p <.05, ^**^p <.01, ^***^p <.001.

The coaching relationship (working alliance) was rated very positively: 96% reported high or very high trust in their Oska coach (range 1–5, *M* = 4.46, *SD* = 0.50). A more positive working alliance was associated with greater perceived stress reduction related to living with CKD (*r* = .43, *p* <.01).

Participants also rated the Oska Program’s contribution to kidney-specific health literacy very highly: 92% considered the program’s impact on their personal health literacy to be high or very high. Correlational analysis showed that the more positively the working relationship was rated, the greater the perceived improvement in health literacy (*r* = .67, *p* <.01).

**Figure 3:**
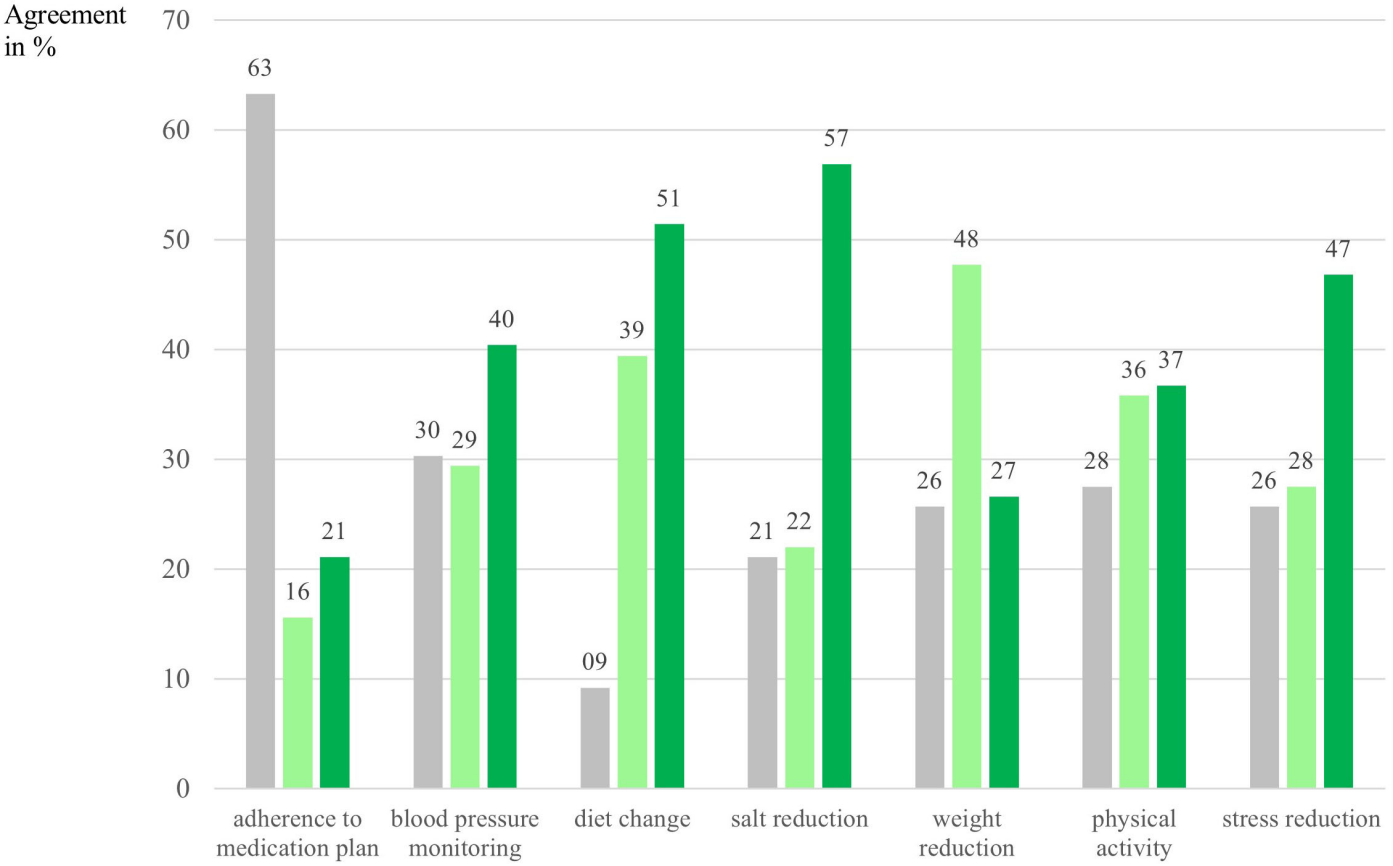
Intention and behavior change per category in percent. *N* = 109. Numbers are depicted in %. Gray bars indicate participants who answered that this category was not applicable/not relevant for them/their condition. Light green bars indicate intenders according to the Health Action Process Approach [HAPA; Schwarzer (35)], meaning that participants had formed an intention to address this particular behavior and had specified goals with their health coach, answer category: “yes, I would like to address that specifically now”. Dark green bars indicate actors according to the HAPA, meaning that participants had already succeeded in implementing changes in this category, answer category: “yes, I already achieved noticeable positive changes with the Oska program”.

In terms of behavior change across CKD-relevant areas, 85% of participants reported having already made at least one positive health behavior change with the support of the Oska program. Most common were: salt reduction (56.9%), dietary changes (54.1%), and reduction of uncertainty and stress related to kidney disease (46.8%), see **Figure 3**. Participants also formed a strong intention regarding weight loss, which might be a more long-term goal, although measurable reductions were already reported. No gender or age differences were observed in overall behavior change, except for slightly better medication adherence in older participants, who also reported taking a greater number of medications, which may have biased these results due to a stronger focus in this area. This category was not relevant for 63.3% of participants, but with higher age and a higher number of chronic diseases its importance increased.

Subgroup analyses showed that participants with a higher BMI were somewhat more likely to implement behavior changes overall, particularly in the area of weight reduction (*r* = .42, *p* <.01). Individuals with diabetes also had a higher likelihood of changing at least one behavior since joining the Oska Program (moderate effect size, Cohen’s *d* = .49).

Having a higher number of chronic diseases was not related to a higher likelihood of behavior change, ratings of working alliance, nor improvements in health literacy. Moreover, the type of insurance (private vs. public) was not associated with any outcome measures; however, this finding should be interpreted with caution, as the proportion of privately insured participants was relatively small. Linear regression analyses investigating the factors contributing to increased health literacy and successful behavior change revealed that greater improvements in health literacy were primarily linked to longer program participation and, above all, a strong trust-based relationship with the coach (large effect size according to Cohen, adjusted *R²* = .48, indicating 48% of variance explained). Successful behavior change (across all guideline-related areas) was most strongly predicted by a positively rated working alliance and perceived contribution of Oska to health literacy—not by sociodemographic factors or the number and type of diagnoses (moderate effect size, adjusted *R²* = .14).

Additionally, overall satisfaction and the likelihood to recommend the Oska program were very high with a Net Promoter Score of 85% and a mean score of *M* = 9.36 (*SD* = 0.83) in the continuous variable, that was used for the correlation analyses. A higher likelihood of recommendation was strongly related to perceiving a positive working alliance (*r* = .49, *p* <.01), a contribution of the program toward higher health literacy (*r* = .59, *p* <.01), and higher achievements in terms of behavior change (*r* = .39, *p* <.01).

### 3.3 Discussion study 2

Analyses of this pilot study indicate that individuals who participated in the Oska program for an average of 4.7 months reported a significant increase in kidney-specific health literacy, and that behavior changes were successfully implemented through structured coaching based on the HAPA model. In particular, the strong emphasis on building a trusting relationship, also known as the working alliance, which is considered a key factor for success in coaching and therapy settings (49, 58), was identified as essential for the development of health literacy, which in turn was the strongest predictor and thus the key to guideline-concordant behavior change.

Our pilot study has several limitations that need to be acknowledged. First, due to our relatively small sample size of *N* = 109 participants, we were only able to detect at least medium-sized effects in regression analyses with up to three predictor variables, whereas for smaller effects and multivariate analyses, our study was underpowered. As this was the first attempt in the field of CKD to study effects of a theory-derived m-health program, the presented preliminary findings require replication in larger samples, which will be done in a larger longitudinal study of the same project. Second, as the study employed shortened and/or adapted scales, the psychometric properties (e.g., reliability, validity) may not fully match those of established instruments. Although internal consistencies were acceptable and adapted items were checked by experts in the fields of nephrology and health psychology for content validity, the interpretability of the findings may be slightly limited due to the pilot character of these measures. Third, self-report measures were used for behavior change data, which is prone to social desirable answers. The initially self-reported comorbidities (e.g., hypertension) were validated by health coaches using physicians’ letters and medication plans uploaded within the Oska app. Objective markers and more standardized and longer measures such as the complete PAM-13 will be analyzed in the ongoing larger study, alongside meta data of the Oska app on participation in webinars, completion of tasks/chapters, and regular entries in diaries (blood pressure, weight, and nutrition diary). Fourth, our study only focused on pre-to-post effects in a within subjects design and lacked a control group of participants not receiving the intervention of the Oska program, which will also be solved with a waiting-control group in the larger ongoing study.

## 4 General discussion and implications

Theoretically derived and evidence-based support programs for self-management in CKD and co-morbidities, including specific training in the form of personal, intensive counseling, are minimal to non-existing in both primary care and clinical settings (18, 59). For individuals with CKD, the complexity of disease-related information, limited awareness and thus impaired health literacy, as well as the absence of easily accessible and understandable information, combined with low self-efficacy, present the greatest challenges (60, 61). Innovative approaches to deliver evidence-based early interventions in a real-world setting are therefore urgently needed to support the self-management of individuals with CKD (62). Our first cross-sectional study among participants with (probable) CKD revealed, that acceptance of an m-health program, which is presented in a short vignette and is offered hypothetically by the health insurance of the target group is quite high, and that the relevant factors for a high usage intention include perceived compatible health benefits, health literacy, and perceived usefulness. In our second study among participants of the Oska program, high satisfaction, very good usability, a strong working-alliance and gains in kidney-specific health literacy were reported. Especially a positive working alliance was predictive for perceived increases in health literacy (large effect size) which was in turn predictive of a higher likelihood for behavior chance (moderate effect size).

A contributing factor to these positive results might be the fact, that the program offered here does not constitute a typical “Digital Health Application” (“DiGA” under the German Digital Healthcare Act); rather, it places particular emphasis on the interpersonal relationship between health coach and participant, which is considered a central component of its intervention approach.

As the trust-based, close relationship between coach and patient is central to Oska’s effectiveness in promoting behavior change, this might raise questions of scalability. However, we argue that this human-centered, but hybrid model can be scaled efficiently. The integration of proprietary digital tools and emerging AI technologies reduces administrative workload and supports coaches in prioritizing meaningful interactions. As our systems have matured, coach productivity has increased, enabling the care of larger patient cohorts without compromising relationship quality. Additionally, Oska offers attractive working conditions in comparison to traditional healthcare settings (i.e., regular hours without night or weekend shifts alongside the opportunity to support patients over a longer timeframe than in short clinic visits), supporting workforce sustainability and retention.

Building on findings by Schmidt et al. (29), who demonstrated that cognitive status predicts performance in everyday technology use among older adults with and without mild cognitive impairment (MCI), future research should include cognitively impaired populations when evaluating digital health programs. Since Schmidt et al. employed performance-based rather than self-reported measures, similar methods could clarify how users actually interact with systems like Oska. A small proportion of Oska participants (not included in the present study), who partly reported mild cognitive impairment, is only coached via landline telephone and receives printed out materials via mail, in an attempt to explore feasibility to also include persons willing to participate but lacking digital skills, a smartphone, or experience digital participation as too stressful due to cognitive issues. Additionally, incorporating social support from family members, peers or trained volunteers may boost motivation and sustained engagement, particularly among older adults managing chronic conditions. This is consistent with Jokisch et al. (28) who highlighted informal and volunteer-based support as key enablers for digital health adoption in later life. This may also represent a promising approach to enhance accessibility for more vulnerable groups, including individuals with limited German language proficiency who are currently not included. Our findings are encouraging regarding user acceptance and initial behavior changes. However, replication in larger, controlled settings is necessary, along with the inclusion of objective indicators to assess both disease-specific outcomes and broader economic implications. If validated in future studies, these results could inform strategies to slow the progression of chronic kidney disease (CKD), thereby alleviating both its financial and epidemiological burden. A recent *Lancet* study highlights the substantial economic impact of CKD, based on a comprehensive analysis across 31 middle- and high-income countries, including Germany, the UK, and the USA (5). The study projects that between 2022 and 2027, annual direct healthcare costs for CKD (prior to kidney replacement therapy) will already increase by 8.7%, with significantly higher costs when dialysis or transplantation is required. Therefore, early secondary prevention programs that employ innovative, low-threshold approaches—such as the Oska Program—are essential to evaluate new pathways for slowing disease progression and reducing the risk of costly complications.

## Data Availability

The datasets analyzed for this study will be available at the open science framework (OSF) when the larger project is completed (https://osf.io/4a3dn/?view_only=ab1c5251a83a49dc9cb3f15dc1acfb2f) and are available upon request from the corresponding author.
